# It’s part of me: autistic adolescents thoughts and feelings towards their autistic identity/autism diagnosis

**DOI:** 10.3389/fpsyt.2026.1767141

**Published:** 2026-08-07

**Authors:** K. E. Sharp, Lauren Baczewski, Jennifer R. Bertollo, Chloe C. Neuman, Andrea Lopez, Elodie Carel, Alyssa Verbalis, Cara E. Pugliese, Lauren Kenworthy

**Affiliations:** 1 Center for Autism, Children’s National Hospital, Washington, DC, United States; 2 Neurodiversity Center for Excellence, Curry College, Milton, MA, United States; 3 Department of Psychology, Loyola University Maryland, Baltimore, MD, United States; 4 School of Education, Indiana University Bloomington, Bloomington, IN, United States; 5 Department of Psychological and Brain Sciences, University of Massachusetts Amherst, Amherst, MA, United States

**Keywords:** adolescence, autism, disclosure/disclose, identity, neurodivergent community, qualitative

## Abstract

**Introduction:**

Adolescence is characterized by active and ongoing identity development. Autism is one aspect of identity and positive feelings about one’s autistic identity predict better self-esteem and psychological wellbeing among autistic people. This study aimed to explore autistic adolescents’ thoughts, feelings, and attitudes about their autism diagnosis.

**Methods:**

Sixty autistic adolescents (Average Age = 16.65 years) in the Washington, D.C. metropolitan area participated in brief semi-structured interviews asking the adolescents to describe their current diagnoses, feelings toward those diagnoses, and whether they disclose their diagnoses to friends or feel comfortable talking about them in social settings. Reflexive thematic analysis was used to code interview responses and generate themes.

**Results:**

From the interviews, five main themes were generated related to participant feelings toward their autism diagnosis: 1) value of neurodivergent community, 2) autism terminology, 3) stigma or stereotypes about autism, 4) journey to understanding, and 5) autistic identity.

**Conclusion:**

This study highlights the diverse, complex, and nuanced thoughts autistic adolescents have about their autism diagnosis. Factors such as internalized stigma, neurodivergent community, and knowledge about autism impact identification with an autism diagnosis. Our findings emphasize the importance of neurodivergent community and neurodiversity-affirming views to increase positive autistic identity and self-acceptance.

## 1 Introduction

Adolescence is characterized by active and ongoing identity development, as individuals begin to make meaning of their lived experiences in the context of their social environment (1). Autism, and disability more broadly, can be a critical aspect of one’s identity that emerges alongside other salient social and personal identities (2, 3). Adolescence often begins this process of self-exploration, although the development of disability-related social identities can span across the life course (4, 5). Despite the clear importance of adolescence as a catalyst for identity development and exploration, much is still unknown about how autistic adolescents feel about their autism diagnoses and disclosure, and whether they consider autism to be a facet of who they are.

Existing research shows that autistic identity development during adolescence can be a complicated process, and adolescents often have mixed, conflicting, and sometimes contradictory feelings about their autism diagnosis (6, 7). For example, in a mixed methods study of 38 autistic adolescents (average age = 15.1 years) by Berkovits and colleagues, many participants listed both negative and positive aspects of their autism diagnosis, but negative statements were more frequent (6). Several of these negative statements related to perceived stigma and fear about how autism would affect their futures. Findings from Jones and colleagues (1) echoed these conflicting sentiments, as autistic young people (ages 13–20 years) described both autistic pride and a desire to reduce social stigma associated with the autism label. Studies show that autistic adults similarly wrestle with worries about negative societal views of autism, acknowledging evident stigma and prevalent stereotypes (8). Stigma can impact one’s self-concept and can act as a barrier to disclosing one’s diagnosis (1, 9). In a study by Frost and colleagues (10), most autistic college students reported they do not disclose their diagnosis to others often; however, those who do disclose choose to tell people that they are close to and can provide them support.

Most prior research on disclosure is focused on the perspectives of parents, caregivers, or other people, rather than the autistic person’s experience (9). Many parents are hesitant to tell their child about their autism diagnosis or to disclose their child’s diagnosis to others, often due to fear of stigma or mistreatment from others (11). However, disclosure to children about their own autism diagnosis is linked to many positive outcomes, such as deeper understanding of personal strengths and challenges, enhanced self-advocacy skills, and more neurodiversity-affirming views (12, 13). Moreover, the viewpoints, terms, and language that others use to describe autism in the presence of autistic young people appears to have significant implications for how a young person views autism, which in turn affects the development of autistic identity (14). When interviewed, parents and teachers explicitly emphasized differences between their autistic children and their neurotypical peers, and often discussed experiences of social camouflaging to fit in. For example, parents often described neurotypical peers as “normal,” reinforcing the othering of the autistic child. Further, some teachers and parents stated that a child is “not *as* autistic” as another student or even doubted a student’s diagnosis because they did not seem “stereotypical[ly]” autistic (14). This rhetoric may suggest to autistic young people that being autistic is negative and may impact disclosure decisions and emerging self-concept.

Fewer studies have explored adolescents’ firsthand perspectives on their disclosure decisions, and what factors may play a role. Extant studies on autistic adolescents’ and adults’ perspectives suggest that many hesitate to disclose or delay disclosing as they weigh perceived pros and cons of disclosure (9, 15). Many people described making deliberate, careful decisions about when to disclose, aiming to minimize stigma and anticipated negative judgment from others (9). Some young people ultimately chose to disclose in order to help others better understand them or as part of reclaiming and embracing their autistic identity (10, 16). Further, some adolescents find other mental health diagnoses easier to discuss than autism (16), and many discuss symptoms of co-occurring conditions when asked about their experiences with autism (3, 6). Taken together, these studies suggest that autistic people are engaged in active discernment processes around whether and when to disclose their autism to others, and these decisions are impacted by factors such as stigma, autistic pride, and who is on the receiving end of that disclosure. In some cases, disclosure may act as an access point to autistic communities, which may lead to a more positive self-view of autism (17).

Overall, research depicts a complex process of socially constructing one’s autistic identity, as self-understanding and meaning-making evolve over time and with changing societal discourse around autism (e.g (5, 14, 18).,). Qualitative studies in Australia and Norway have shown how autistic adults came to understand and accept their autism through a lengthy process of many years, with often positive and empowering results (18, 19). This idea aligns with existing models of Disability Identity Development, which describe identity conceptualization and integration as ongoing across the life course (4). Gobbo & Shmulsky (5) applied Gibson’s model of Disability Identity Development to autistic people, positing that autistic identity development occurs in three general stages (1): In *passive awareness*, typically during childhood, a person does not identify with autism and is often influenced by cultural views and ableist ideologies (i.e., beliefs and practices that discriminate against people with disabilities (20)) (2). *Realization* often occurs during adolescence or early adulthood, and is marked by emotional distress (e.g., self-hate, denial of diagnosis, rejection of negative messages about autism) (3). *Acceptance* is usually reached in adulthood, when individuals may embrace their differences, seek neurodiversity-affirming community, partake in disability-related social advocacy, and otherwise integrate more authentically into society. Recent studies of autistic adults illustrate that many describe their autism in a more neutral manner (18), aligning with the *Acceptance* stage of this model. In recent years, societal views of autism continue to evolve with greater visibility of neurodiversity-related language and approaches, which view autism as an identity and culture, while simultaneously acknowledging more challenging aspects of being disabled (21)). In the context of these shifts in societal discourse around autism and a greater understanding of autism as an aspect of identity, it is critically important to examine the firsthand perspectives of autistic adolescents as they make sense of their autism diagnoses and identities.

Understanding adolescent identity development around autism and disability is relevant to both individual understanding and long-term outcomes of autistic adolescents. A strong, positive autistic identity is linked to better mental health, wellbeing, and self-esteem among autistic young people and adults (22, 23). For example, in a sample of 122 autistic young people (ages 15–22 years), higher autism satisfaction was associated with higher psychological wellbeing and lower social anxiety, emphasizing the connection between autism social identification and mental health (23). Positive autistic self-concept may act as a buffer against the increased mental health risks of autistic people (24). Having a strong affiliation with one’s autistic identity is also related to more disclosure, which in turn is linked to fewer camouflaging behaviors (25). As the link between a positive autistic identity and downstream mental health outcomes becomes increasingly clear, it is important to gain a greater understanding of adolescents’ understanding of their autism.

### 1.1 Current study

The current study explored autistic adolescents’ thoughts, feelings, and attitudes about their autism diagnosis and identity via a brief semi-structured qualitative interview. Specifically, this qualitative study aimed to understand how they feel about their autism diagnosis and whether adolescents disclose their autism diagnosis to friends or peers. Adolescents’ interview responses were coded, and themes were generated using Braun and Clarke’s reflexive thematic analysis (26). Themes are described along with exemplar quotes and relevant clinical implications for the autistic community are discussed. Emergent themes and interpretations are provided within the context of the authors’ identities and positionalities.

## 2 Materials and methods

This study was part of a larger clinical trial evaluating mechanisms associated with learning and cognitive flexibility in high school-aged autistic youth, which received approval from the Children’s National Institutional Review Board (Pro00015492). Study participants received a group-based executive function intervention for autistic adolescents. Data were collected across three waves of intervention groups between 2022-2025. Participants took part in a brief pre-intervention intake interview to collect information pertinent to therapeutic group placement (described further in sections that follow). We conducted a qualitative reflexive thematic analysis (RTA) of the two questions of the semi-structured interview that were related to autism diagnosis and feelings regarding disclosure. Specifically, these items inquired about participants’ feelings about their autism diagnosis and their willingness to disclose their diagnosis to friends or peers.

### 2.1 Participants and eligibility

Participants were recruited through clinical and research databases, online listservs, social media, schools, community centers, and referrals from local providers. Participants were 60 adolescents ranging in age from 14.77 to 18.57 years old (*x̄* = 16.65) at the pre-intervention interview study visit. To be eligible for this study, participants needed to have a formal autism diagnosis or an educational autism classification such as autism being listed on an Individualized Education Program (IEP) or 504 plan, as reported by parents/caregivers. Participants provided their most recent psychological evaluations (if available) and the research team reviewed them to confirm an autism diagnosis or classification. If documentation was not available, a clinical psychologist with expertise in autism confirmed that participants met Diagnostic and Statistical Manual-5-Text Revision (DSM-5-TR (27)) criteria for autism, supported by the use of the Autism Diagnostic Observation Schedule-2 (ADOS-2 (28)), parent report of lifetime and current traits/characteristics, review of records, and their clinical judgement.

The majority of youth in this sample were White (*n* = 47, 78.3%), not Hispanic/Latino/Latine (*n* = 54, 90%), and male (*n* = 42, 70%). There were 11 gender-diverse youth participants (18.3%) who self-reported a gender different from their assigned sex at birth. In addition to autism, adolescents in this sample had various co-occurring mental health or behavioral conditions such as attention-deficit/hyperactivity disorder (ADHD; *n* = 39, 65%), anxiety (*n* = 16, 26.7%), and depression (*n* = 5, 8.3%). Participants had abbreviated full-scale intellectual quotient (FSIQ) scores in the average range (*x̄* = 109.62, *SD* = 13.44, Range = 83-142), as measured by the Matrix Reasoning and Vocabulary subtests of the Wechsler Abbreviated Scale of Intelligence-II (WASI-II (29)), or equivalent subtests on the Wechsler Intelligence Scale for Children-V (WISC-V (30)) or Wechsler Adult Intelligence Scale-IV (WAIS-IV (31)). At the time of the interview, the Social Responsiveness Scale-2 (32) was completed by caregivers to quantify and characterize their child’s autism traits, where adolescents had a mean *T*-score of 71.78 (*SD* = 10.82, Range = 45-100), which is within the ‘moderate’ range. See **Table 1** for adolescent demographics.

**Table 1:** Adolescent demographic characteristics.

Characteristic	*n*	*%*
Latine identity		
Yes	6	10
No	54	90
Ethno-racial identity		
American Indian, Native American, Indigenous	1	1.67
Black	6	10
Black & Indigenous	1	1.67
Asian	1	1.67
White	47	78.3
Asian & White	4	6.7
Identify as gender diverse?		
Yes	11	18.3
No	49	81.7
Gender identity		
Male/man	42	70
Female/woman	13	21.67
Nonbinary	4	6.67
Prefer not to say	1	1.67
Sex assigned at birth		
Male	42	70
Female	18	30
Other behavioral/mental health condition^a^		
Attention-Deficit/Hyperactivity Disorder	46	76.67
Specific Learning Disability	17	28.3
Adjustment problems	6	10
Anxiety	45	75
Conduct problems	4	6.67
Depression	28	46.67
Disruptive behavior problems	12	20
Eating disorders	1	1.67
Trauma	1	1.67
Psychosis	2	3.33
Other	1	1.67
None	4	6.67
Study cohort		
Year 1	20	33.3
Year 2	19	31.7
Year 3	21	35
Age in years at interview (M, SD)	16.65	1.18
Full Scale IQ (M, SD)	109.62	13.44

^a^ Participants can be included in more than one category; therefore, n > 60.

### 2.2 Procedures

Prior to beginning the intervention program, adolescents met with clinical research coordinators and completed a brief intake which included an introduction to the intervention and a semi-structured interview (see details below).

#### 2.2.1 Part 1: demographic survey

Participants and their caregivers responded to a demographic survey during the interview study visit. Demographic data (see **Table 1**) reflect parent/caregiver-reported demographics, except for gender identity which was participant-reported.

#### 2.2.2 Part 2: brief semi-structured interview

The pre-intervention intake interview probed for information relevant to therapeutic group placement. The intake interview was designed to confirm the adolescent’s interest in participating in the group intervention while gaining insight into some of their personal experiences and goals. To begin, participants were asked how they get along with others their age, their favorite subject in school, and to describe some of their goals in life. The remaining two interview questions asked participants about their autism diagnoses. Prior to asking these two questions, research coordinators read participants the following statement: “In the group, we will talk about self-advocacy, neurodiversity, and thinking styles. As part of this discussion, some group members find it helpful to discuss their diagnoses and enjoy being part of a community, while others prefer not to talk about it.” Next, participants were asked: “Do you have any current diagnoses? For example, autism, ADHD, or learning disabilities?” and “What do you think about your diagnoses?” The interview was designed in collaboration with several clinical psychologists, some of whom have implemented the intervention in prior years and found these questions to be clinically relevant. During the course of the study, the team added an additional question about disclosure and 36 adolescents answered this additional question: “Do you tell your friends about your [diagnoses]?”

Interviews took approximately 15-25 minutes and were administered by two research coordinators who were involved in the creation of the interview questions. Adolescents were interviewed at the end of their pre-intervention appointment, after interviewers had established rapport with the participants. Participant responses were transcribed verbatim by the research coordinator during the interview.

### 2.3 Data analysis

We used Braun and Clarke’s approach to reflexive thematic analysis (RTA) to explore the thoughts and feelings of autistic adolescents about their autism diagnosis (26). We aimed to center participants meaning making in the data, and the first-level codes best represent those perspectives. Our team held a critical realist lens—and thus we viewed participants’ responses to be shaped by their individual experiences of autism, while acknowledging that some degree of subjective reality may simultaneously exist (33). Our analysis of the data was guided by our team’s alignment with the neurodiversity paradigm (21), which views autism as an identity and culture.

**Figure 1:**
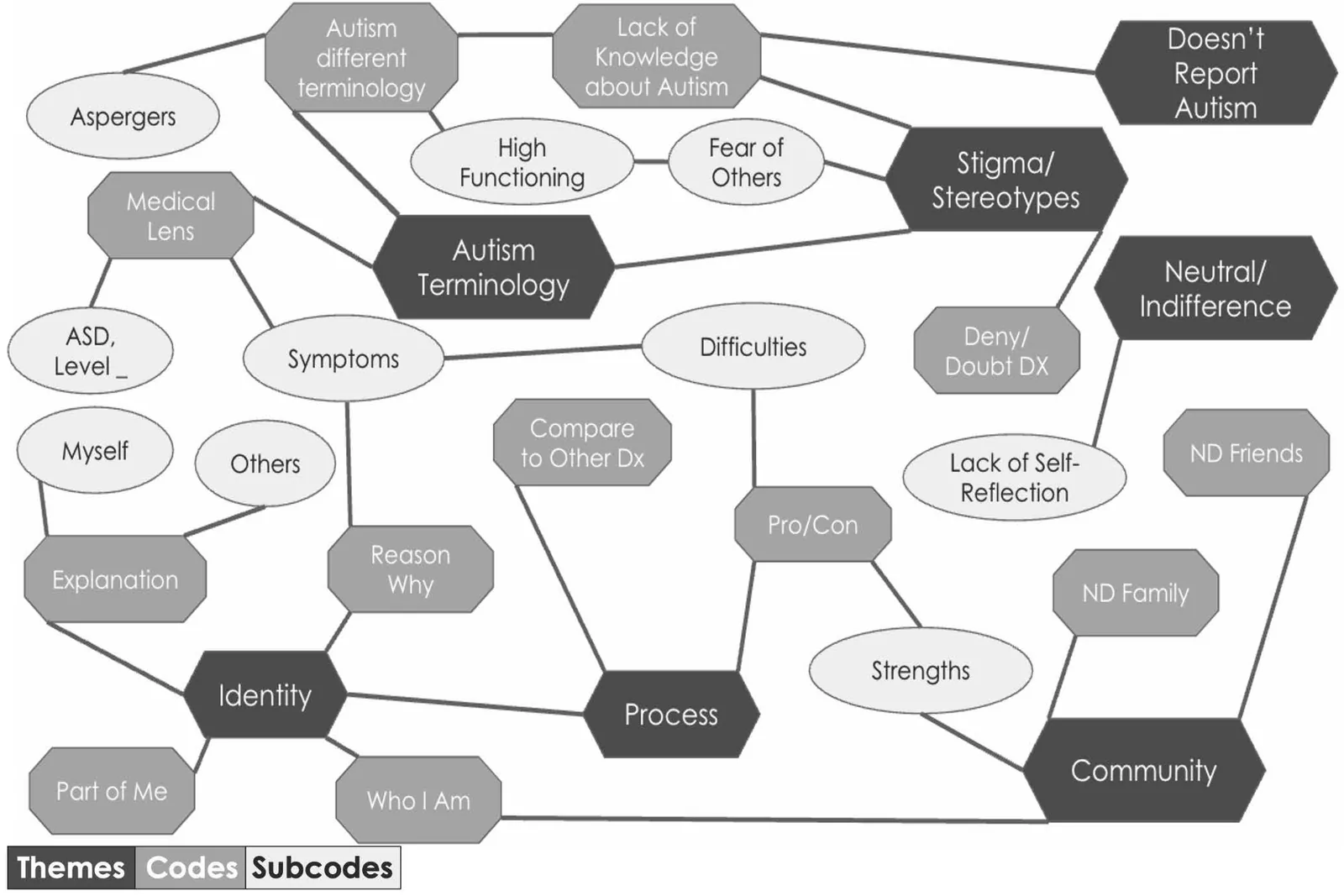
Preliminary thematic map. Dark gray shapes represent candidate themes, medium gray represents candidate codes, and light gray represents candidate subcodes. Lines between shapes indicate relationships between the codes and themes. This thematic map reflects the early meaning-making process based on participants’ responses and how these concepts relate to one another, not the final themes and codes. “ASD Level”, autism level as categorized in the Diagnostic and Statistical Manual of Mental Disorders, 5th Edition (DSM-5).

We analyzed the data using the procedural steps of RTA (26). The first four authors coded and analyzed the data. First, we collated written interview transcripts and familiarized ourselves with the data by reading through all participant responses to the two selected interview questions. Next, we coded interview responses inductively based on repeated patterns in the data, and generated preliminary themes related to autism diagnosis and disclosure (see **Figure 1** for preliminary codes, subcodes, and themes which were continuously refined). We met multiple times to discuss these preliminary themes and further develop, refine, and name them. We combined several codes and initial themes from this map into a total of five final themes.

Our analysis was shaped by the positionalities and experience of our research team. In RTA, researcher subjectivity is seen as an asset to data interpretation, and reflexivity is seen as a tool to more deeply understand the desired phenomenon (26). Throughout our analytic process, the first four authors shared their individual perspectives on the data based on our lived experiences. One of the four coders identifies as autistic, one identifies as neurodivergent but not autistic, two have an autistic family member, and all four coders have extensive clinical and/or research experience working with autistic adolescents and young adults. These experiences influenced our interpretation and meaning making of the data.

## 3 Results

Five main themes were generated related to participant feelings toward their autism diagnosis: 1) value of neurodivergent community, 2) autism terminology, 3) stigma/stereotypes about autism, 4) journey to understanding, and 5) autistic identity (see **Table 2**). Exemplar quotes for each theme are followed by participant ID numbers. Sixty-four percent of adolescents who completed a question about disclosure (23/36) reported a willingness to tell their friends about their autism diagnosis. Although all participants had a parent/caregiver-reported autism diagnosis, 15% (9/60) of adolescents did not report autism when asked to list their diagnoses, even when the interviewer explicitly named ‘autism’ as an example diagnosis.

**Table 2:** Final themes with sample codes and quotes.

Theme	Sample codes	Exemplar quotes
Value of Neurodivergent Community	· Neurodivergent Friends · Family	“It’s like the sky is blue, I have other neurodiverse friends, so it’s just normal”
Autism Terminology	· Aspergers · ‘High Functioning’ · Medical Terminology	“I don’t really consider myself autistic, I think maybe I’d say high functioning autism”
Stigma & Stereotypes	· Difficulties · Fear of Others · Denial/Doubt of Diagnosis	“I don’t think I’m autistic, I am just lazy sometimes.”
Journey to Understanding	· Weighing Aspects of Diagnosis · Comparing to Other Diagnoses · Pro/Con	“I feel there are more pros about autism and ADHD than there are about OCD”
Autistic Identity	· Reason Why · Explanation to Myself and Others · Part of Me · Who I Am	“It’s just who I am. Plus if I didn’t tell people about them, they’d just think I’m insane. Like no, there is a reason I’m like this, and I’m kind of glad there’s a reason.”

### 3.1 Theme 1: value of neurodivergent community

Many participants reported having neurodivergent friends and/or family members. Having connections to other neurodivergent people allowed adolescents to feel more comfortable talking about their autism diagnosis with others. For example, one participant shared, *“I have a lot [of] online friends that also have some diagnoses, so that has helped a lot”* [Participant 22]. When asked about disclosure to friends or peers, one participant shared that they choose to disclose specifically to those within the neurodivergent community, saying *“…sometimes I talk about them [my diagnoses; dyslexia, ADHD, autism] with people who also have them”* [Participant 45]. For several adolescents, having neurodivergent friends made them feel more connected to others in their lives and ‘normalized’ their autism and co-occurring diagnoses. One participant described this sentiment, *“It’s like the sky is blue, I have other neurodiverse friends so it [my autism diagnosis] is just normal”* [Participant 36].

Some reflected on how connection to the neurodivergent community impacted their sense of belonging and identity. For example: *“…in some ways I’m proud of the ASD diagnosis. I’m part of the ASD community. I’m happy being autistic, I don’t mind that at all*” [Participant 65]. Many reflected that they gravitate toward other neurodivergent and/or autistic people in their peer group or neurodivergent people in their family. They found it easier to relate to others who shared their diagnoses/neurodivergent identity and had similar lived experiences, as one participant remarked, *“…[my autism] makes me relate to my dad*” [Participant 31]. Many of the adolescents who chose to disclose their autism diagnosis to friends/peers simultaneously mentioned having neurodivergent friends. Taken together, participant reflections suggest that connecting with other neurodivergent people may have impacted their decisions to disclose and aided in self-acceptance and comfort with their own diagnoses.

### 3.2 Theme 2: autism terminology

Participants used a range of terms when talking about their autism diagnoses and other disabilities/diagnoses. While many adolescents used the term ‘autistic’, others used different words to describe their autism (i.e., “Aspergers,” “autism- type 1,” “ASD,” “high functioning autism”). Several adolescents commented on how autism-related terminology has changed across the course of their life and how language will continue to shift in the future. Changing autism terminology seemed to impact adolescents’ understanding of their own diagnosis– and in some cases left them feeling confused about the differences between terms or uncertain of the words that fit them best. Participant 6 reflected: *“When I was younger, I was diagnosed with Asperger’s, but since it’s not a thing anymore, I’m not sure what I am anymore, I think it’s autistic, but I don’t really consider myself autistic. I think maybe I’d say high functioning autism.”* This adolescent and several others identified with terms like “high functioning” or “autism-type 1.”

In some cases, adolescents seemed to want to distance or differentiate themselves from other autistic people (particularly those with higher support needs) and chose to use functioning-label terms to create or emphasize this distance. Implied in this distance was the notion that autism was a hierarchical continuum, which Participant 50 echoed when asked about the diagnoses he had and reflected, *“I’m pretty sure [I have] autism, but I’m not sure”* and later, when asked what he thought about his diagnosis: *“It’s at the bottom of the scale so it doesn’t really count. I don’t know. People have autism. They’re going to change the name in the DSM-VI.”* Another adolescent preferred the term ‘Aspergers’ to describe his autism, not to distance himself from other autistic people, but because he felt the history of the term was important: *“…I disagree with the censoring of Asperger’s so I still call it Asperger’s syndrome because I don’t believe in censoring history.”* [Participant 53].

Overall, participants used a diverse range of terms to describe their diagnoses, indicating that they hold diverse views of autism. Some expressed more explicit preferences for medicalized terminology, while others’ preferences were shaped by their knowledge of autistic history, or simply the terms they had heard used around them. Some adolescents used terms interchangeably (e.g., ‘ASD’, ‘autistic’), while others listed two distinct terms for autism as if they were separate diagnoses (e.g., *“[I am diagnosed with] Asperger’s and autism”* [Participant 17]). There appeared to be large variation in the meaning that adolescents attributed to different autism-related terms rather than a shared understanding of the core features or traits associated with autism.

### 3.3 Theme 3: stigma and stereotypes

When asked to share thoughts about their diagnoses, many adolescents shared experiences with autism-related stigma, both internalized and externalized. Several participants acknowledged stigma toward autism as a reality of the world and expressed fear of judgement or other negative experiences because of their diagnosis. One adolescent described this apprehension, saying: “*I just hope that getting them [autism, ADHD, dyslexia] diagnosed will actually help me instead of hinder me. Because sometimes you might be restricted or looked down on*” [Participant 45]. When asked about their comfort in disclosing their diagnosis, another reflected, “*…if I didn’t tell people about them [autism, ADHD, generalized anxiety disorder, depression], they’d just think I’m insane*” [Participant 55].

Descriptions and examples of internalized stigma toward autism were often shared by participants. There were many more examples of internalized stigma than externalized stigma experiences. One way that internalized stigma was evident in participant responses was through some participants’ clear aversion to or rejection of their autism. In sharing their thoughts about their diagnoses, one participant stated that they “hate” their diagnoses (including autism, ADHD) [Participant 54], while participant 46 reflected, “*I don’t know about my autism– I feel like my genes screwed me over.*” When given autism as an example of a diagnosis one might have, participant 37 responded, “*I am normal, fuck you, no*”. Echoing these sentiments, one adolescent reflected, “*honestly, I wish I didn’t have them* (Autism, ADHD) [Participant 59]. Other adolescents expressed some degree of shame about their autism diagnosis: “*I feel a little different about it, ashamed about it*” [Participant 5].

Some adolescents described attempting to distance themselves from some aspects of autism or distinguish themselves from other autistic people. In these instances, internalized stigma about what it means to be autistic, as well as stereotypes about what autism “looks like,” impacted adolescents’ descriptions of how they relate to their autism. Participant 32 reflected: “*I think when I was diagnosed [with autism] they [providers] messed up. I don’t think I’m autistic I am just lazy sometimes, I get social cues but I choose to ignore them…”* Another adolescent created distance between themself and other autistic people when saying, “*…when I hear autism, I think severe autism, and I don’t want people to see me that way. I don’t feel like I am* ***that*** *autistic*” [Participant 48].

While many adolescents shared reflections that showed evidence of autism-related stigma, several participants described additive experiences in which autism enriched their lives (e.g., connection to autistic community or identity; as described in Themes 1 and 5).

### 3.4 Theme 4: journey to understanding

Participants processed or attempted to make sense of their autism diagnosis in a variety of ways. Some adolescents indicated less self-reflection or a lack of understanding about autism, several participants compared their autism to their co-occurring diagnoses, and others weighed pros and cons of their diagnosis. Many described gaining knowledge about and understanding of autism over time.

Several participants shared that they were unsure or had no substantive comments or reflections related to their autism diagnosis. When asked to share thoughts about their autism, participant 7 responded, “*Meh, never really thought about it*.” Some participants expressed their lack of knowledge about what their diagnosis meant, with participant 11 sharing “*Asperger’s, but I don’t know what it is or what it means.”* Other participants were actively engaged in a process of understanding their diagnosis but still lacked accurate information about autism and expressed a desire to learn more. Participant 57 shared, “*Right after I had my MRI, I asked my mom what autism is, and she told me it is a disease … What is autism [question posed to interviewer]?*” Participant 13 began describing their autism in the context of their ADHD diagnosis, and expressed a general lack of knowledge about autism and disability:

*“I have ADHD. My dad said I have a lot of it. I’ve known I had ADHD for a while, and I know I can’t change that. Well actually I think I have ADD, not ADHD. I’m pretty comfortable talking about it. I’m not embarrassed. I think if it was more personal like autism, I mean I know it’s common, but I think that feels a little more personal. I’ve heard people call people retarded … what is the best way to call someone that? Because I know that’s rude. Is being retarded a part of autism?”*

Given that one of the interview questions asked participants to describe their feelings about all their diagnoses, many participants shared their thoughts on diagnoses other than autism. Those who had co-occurring diagnoses reflected on their autism in relation to these other diagnoses. For example, one participant described, “*there are more pros about autism and ADHD than there are about [obsessive compulsive disorder (OCD)].*” [Participant 63] and another reflected, “*…I’m happy being autistic, I don’t mind that at all. I wish I didn’t have ADHD, depression, or anxiety though*” [Participant 65].

Many adolescents felt that their understanding and acceptance of autism grew over time:

*“Autism, I kind of grew to understand it. People with autism seem to be better at things, like Bill Gates, Stevie Wonder, Albert Einstein. So, I think it’s like a superpower. What I don’t like is my OCD, other than that everything’s fine”* [Participant 64].

One participant’s reflection on their journey to understanding is illustrative of the many different reflections that other participants shared, as it illustrates: weighing and wrestling with autism in comparison to other diagnoses, change over time in feelings toward diagnoses, and learning to understand autism. They ultimately concluded that many of their strengths (e.g., in animation and art) stemmed directly from their autism:

*“It took me a little while to come to grips almost? With the autism diagnosis. Some days it can be easy to slip back into that mindset of “why do I have this thing? God.” But here’s the thing, I don’t think I would be as passionate about animation and wanting to be a board artist without the autism. The ADHD diagnosis I’m more mixed on. I view the autism somewhat more positively? I don’t want to say I can’t think of a single positive thing about the ADHD but … This is hard. I feel like the ADHD feels more debilitating? At least unmedicated, it feels more draining. The autism can be draining too, but the ADHD I don’t feel like there are enough positive things to drown it out. I also think I have OCD and I* *hate* *that, that is 10x worse than either of the others.”*

Participant 58’s reflection illustrates the nuanced process of navigating mixed feelings about an autism diagnosis, while beginning to uncover how autism shapes aspects of who they are (i.e., their interests and aspirations).

### 3.5 Theme 5: autistic identity

Participants’ level of identification with autism as an aspect of identity varied. For those who strongly identified with autism as an integral aspect of who they are, autism was described as shaping and influencing their interests, passions, and sense of self. One adolescent reflected, “*Autism is like, I don’t think I would literally be myself without it.*” [Participant 63], while another saw autism as their “*superpower*” [Participant 64], giving them unique skills, creativity, and perspective that not everyone has.

While some felt very strongly about autism as a core aspect of their identity or sense of self, others had neutral or indifferent feelings toward their autism. For these individuals, autism did not have an out-sized impact on their life relative to other aspects of their identity or experiences. One adolescent reflected, “*I feel like it [my autism diagnosis] doesn’t define me, I’m fine with it and it’s there, and knowing I have it helps me pinpoint my struggles*” [Participant 48]. Another shared similar sentiments, “*[My autism] is part of me. I can always talk about it. I don’t see it as a liability or the only thing in my life*” [Participant 56]. Many others echoed this idea, noting that autism is “*just who I am*” [Participant 44, Participant 55].

When reflecting on their autistic identities, several participants remarked that they always felt ‘different’ from others around them. Growing up in a neurotypical-dominant society, many felt that getting an autism diagnosis helped them to understand themselves more. One adolescent reflected, “*I have always known I was different and now I have a reason why*” [Participant 18]. Others remarked that receiving an autism diagnosis helped them explain their differences or traits to other people. Without this explanation, these participants expressed concern or apprehension about the conclusions that people might draw (e.g., being seen as ‘crazy’ or ‘weird’): *“Plus, if I didn’t tell people about them [my diagnoses], they’d just think I’m insane. Like no, there is a reason I’m like this, and I’m kind of glad there’s a reason”* [Participant 55]. This sentiment was echoed by another adolescent who shared, “*I let certain people know so that if they see me doing something a bit weird, they know it’s because of my disability.*” [Participant 45].

Taken together, findings suggest that adolescents had varied ways of relating to their own autism and different degrees of connection to their autistic identities. For many, receiving an autism diagnosis gave them language to describe some of their traits to others and gave them a new window into understanding themselves.

## 4 Discussion

This study explored autistic adolescents’ thoughts, feelings, and attitudes toward their autistic identities and diagnoses using reflexive thematic analysis of brief semi-structured interviews. Findings shed light on autistic identity development during adolescence—a critical life-stage in which identity formation is active across several axes of identity (34, 35). Overall, adolescents in this sample described nuanced, and at times contradictory, views toward their autism diagnoses. Participants weighed both positive and negative aspects of their autism and many shared neutral feelings toward being autistic (e.g., “It’s just part of me, it doesn’t define me”). Understanding one’s autism diagnosis was a journey that took place across years, and self-acceptance often came with time. Connection to other neurodivergent individuals aided participants in their understanding of what it means to be autistic and appeared to encourage the development of a positive self-concept in relation to autism. Stigma toward autism was salient throughout participants’ responses, as evidenced by their use of socially and clinically outdated disability-related terminology and descriptions of autistic stereotypes. Many participants also expressed a lack of knowledge about autism more broadly or did not report autism as one of their diagnoses. In the sections that follow we integrate these findings with existing research, highlight next steps for future studies in this area, and provide recommendations for caregivers and clinicians who support autistic individuals.

Participants described engaging in varied amounts of self-reflection about their autism diagnoses. For example, some participants discussed not having ever thought about their autism diagnoses, others shared that they hated the diagnoses, while still some expressed that they had grown to understand and accept the diagnosis, and were even proud of it. Beyond taking time to understand their diagnosis, participants discussed how their diagnosis helped them understand themselves. This range in perspectives, and especially the reflection on how feelings toward their diagnosis shifted over time, is in line with previous literature on autistic adults and their autistic identity development (18), and theories on disability identity development that illustrate that feelings about one’s disability identity change and grow across developmental stages (4).

### 4.1 Identity development

Participants’ reflections indicated that not all adolescents in this sample were in the same stage of identity development/conceptualization, despite being in the same broad age group. While Gibson’s (4) model of Disability Identity Development proposes that the three stages of disability identity development are aligned with life stages (i.e., childhood and passive awareness; adolescence and realization; adulthood and acceptance), adolescents in our sample appeared to be actively engaged in each of these three stages. We may see all stages of disability identity development represented in our sample because adolescents are actively participating in a construction of identity, more broadly and in their relation to autism specifically, that is inherently nuanced and non-linear. While studies in adults show varying levels of autistic identity integration, most adults in these studies identify positively or have neutral feelings toward their autistic identity (8, 36), which would be consistent with the notion that acceptance comes with time. Our findings indicate that there may be other factors besides age alone that may influence where adolescents are in their disability identity development, such as time since receiving diagnosis, or the adolescent’s understanding of autism.

Our participants discussed both positive and negative aspects of autism, often together, or shared net neutral descriptions about autism. This is consistent with previous findings that most autistic people describe both positive and negative feelings toward their autism (3, 6, 16, 37–39). For example, Berkovits and colleagues (6) determined that 97.4% of autistic adolescents identified at least one negative element of autism (e.g., social difficulties; restricted, repetitive behaviors and interests) and 76.3% generated at least one positive statement (e.g., cognitive benefits, having a different perspective). The neutral descriptions from many of our participants give credence to the findings of studies that examine autistic adults’ feelings toward their diagnoses (8). Taken together, our findings coupled with those of Botha and colleagues (8) suggest that autistic people of multiple ages acknowledge both positive and negative experiences related to their autism, which is consistent with the neurodiversity model, rather than the inherently negative characterizations found within medical models (40). Participants, in discussing the positive, negative, and neutral aspects of autism, make the case that autism is not a net good or bad; rather, it is a complex identity to navigate with many participants demonstrating a wide range of relationships with this aspect of their identity.

Participants discussed the pros and cons of autism not only on its own, but in comparison to their other co-occurring diagnoses. This finding is consistent with results of extant studies of autistic adolescents, wherein many participants discuss symptoms of co-occurring conditions when asked about their experiences with autism (3, 6). When discussing autism, many participants explained the positives and negatives in juxtaposition to another diagnosis, such as autism being more “personal” than ADHD, or autism being something they would not like to change versus not wanting to have OCD.

### 4.2 Value of neurodivergent community

For many of our participants, connection to other neurodivergent individuals shaped how comfortable they were talking about autism and impacted their decisions to disclose their autism diagnosis to others. Participants discussed sharing their diagnosis with others who had the same diagnosis or were more broadly neurodivergent. This is consistent with findings that autistic individuals disclose their diagnoses to people who are close to them and can provide them with support (41), and tend to gravitate toward others with shared identities and lived experiences (42). Participants discussed the presence of autistic and neurodivergent friends and family as normalizing autism for them, potentially contributing to feelings of safety, self-acceptance, and a greater willingness to disclose one’s diagnosis. Research shows that both autism acceptance and positive autistic social identity are linked to better mental health (43, 44). Our findings suggest that presence of a neurodivergent community may be one avenue to support autism acceptance and identity development, and by extension, positive mental health. Given that autistic individuals experience substantial minority stressors and mental health challenges (8, 24), access and connection to neurodivergent communities may act as a protective factor for adolescents undergoing active identity development and conceptualization.

### 4.3 Stigma and autism knowledge

Some participants felt the pressure of stigma from others and described using disclosure as a form of stigma management. Participants discussed how telling others that they were autistic gave a reason for their behavior rather than letting others draw their own conclusions about them. This is consistent with findings that autistic individuals at times make deliberate decisions to disclose to avoid negative perceptions from others or as an explanation to be better understood by others (9, 10). While this is one approach to stigma management, other studies have acknowledged the presence of a “double bind,” where autistic individuals suffer consequences both for disclosing and for not disclosing (8). Our participants acknowledged this as well, sharing that they hoped the diagnosis would help them, rather than hurt them.

Beyond concerns of external stigma, our participants also described their experiences with autism in ways that showed evidence of internalized stigma. Participants discussed some negative views of autism, and at times used negative or stereotyped language to describe themselves or the autistic community. Internalized stigma and stereotypes about autism and disability appeared to impact participants’ acceptance and level of identification with autism. Our participants described internalized stigma more often than externalized stigma, which differs from the stigma-related experiences of autistic adults, who describe more explicit worries about discrimination and oppression from other people (8). Much of the internalized stigma was evident through an aversion to or rejection of autism (e.g., Participant responding “I am normal, fuck you, no” when asked about their autism diagnosis). Participants also showed evidence of negative stereotypical beliefs about autism, such as that autism was associated with insanity, weirdness, laziness, or “retardation”. These stereotypes are prevalent among non-autistic individuals as well (45, 46), implying that autistic young people are not immune to similar internalizations of inaccurate messages about autism.

Evidence of internalized stigma was also seen in some adolescents’ usage of outdated or ableist terms to refer to autism (e.g., “Aspergers,” “high functioning,” “severe autism”). This finding is consistent with some studies, where some autistic individuals have continued to use their Asperger’s or ‘aspie’ labels (47). Among those who were previously diagnosed with Asperger’s, concerns include losing access to a diagnostic label and related supports, increased stigma from sharing a diagnostic category with high support needs autistic people, and losing a core part of their identity (48–51). Our findings reflect similar concerns. One of our participants reported their reasoning for continuing to use the Asperger’s label was to avoid censoring history, whereas other participants made sure to designate that they were ‘high-functioning’ and not like other autistic people.

Some participants discussed autism as a hierarchical characterization or on a continuum, saying they were not *that* autistic, not *as* autistic as others, or not *severe*, which contrasts findings in a similar study in adults, where participants described autism as a discrete category of autistic or not autistic (8). However, these hierarchical descriptions of autism are consistent with DSM-5-TR autism levels (27) and prior findings on the language used by some parents and teachers (14). Autistic adolescents may be hearing this medicalized language used by parents and teachers and echoing that language when discussing their own autism. Our participants discussed their own lack of knowledge about autism, sharing what they had been told by others (e.g., their parents) and asking the interviewer if these things were true. Participants asked if autism was a disease or if “being retarded” was a part of autism. While there is less research that specifically evaluates the level of understanding that autistic adolescents’ have about autism, studies on the general population found that while there is high awareness of autism (52, 53), misinformation about autism is still prevalent (54). In line with findings in the general population, our participants appeared to have critical gaps in knowledge about the details of autism and expressed a desire to know more.

Beyond misinformation and gaps in knowledge about autism more broadly, some adolescents did not report autism as one of their diagnoses. When asked about their diagnoses and given an example of autism as one such possible diagnosis, many adolescents (15%) in this sample did not report autism. This was unexpected, as all participants had a parent-reported medical autism diagnosis or educational autism classification. There are multiple possible reasons as to why a participant might not have chosen to share about their autism diagnosis. For example, they might not have felt comfortable sharing their diagnosis with the interviewer, or they may have forgotten to mention this diagnosis. Previous studies have found that autistic adolescents may not spontaneously state autism as part of their identity (13, 55). However, considering that our interviewers gave an explicit example of autism as a diagnosis in the question, it is also possible that some participants may not have known about their autism diagnosis at the time of the interview.

The possibility that some of our participants may not yet know about their diagnosis would align with previous findings of a “disclosure delay” between when a child is diagnosed and when they are told about their diagnosis (15). This is crucial to consider, as disclosing an autism diagnosis to a child leads to more positive outcomes (12) and a higher likelihood of neurodiversity-affirming views (13). Considering that participants’ acceptance toward a diagnosis appeared to develop over time, this disclosure delay may also delay the process of disability identity formation.

### 4.4 Implications: importance of autism education and community for autistic adolescents

The results of this study highlight the range in feelings and amount of knowledge that autistic adolescents have about their autism diagnoses. While some felt that autism was a core and celebrated aspect of who they are, others did not identify as autistic despite having a documented diagnosis. Some adolescents expressed confusion about what autism is and used outdated and sometimes derogatory terms to refer to autistic people. These findings highlight a need for greater education about autism for adolescents, led by or including autistic adolescents.

#### 4.4.1 Recommendations for families

Considering that acceptance of one’s own autism develops over time, knowledge about a diagnosis is an important step in developing positive autistic identity and self-understanding. Our findings suggest that autistic individuals should be told and educated about their autism diagnosis. Earlier disclosure about a diagnosis starts the clock on identity formation and self-acceptance and diagnoses also often serve as an access point to autistic communities (17), which may be a protective factor against minority stress. Families should encourage connection with neurodivergent communities, which can be found through family, school environments, friends, and online communities.

When discussing a diagnosis, caregivers should also consider the language and messages they convey about autism, as this language may be internalized and echoed later. It can be helpful to keep this conversation positive, truthful, and personalized to their child’s needs (11, 56). Overall, caregivers can support the development of positive and neurodiversity-aligned views of autism and self-identity by intentionally discussing autism with their autistic youth early on and with neutral or positive language (13).

#### 4.4.2 Recommendations for clinicians and other professionals

Given that many participant reflections indicate a level of internalized shame or stigma about autism, clinicians working with autistic adolescents should engage in self-reflective practices to evaluate how internalized ableism impacts their practice and interactions with clients. Others have echoed similar recommendations for providers (57, 58). Clinicians should seek to directly address the root of such messaging and encourage positive associations with autism and accurate understanding of the autism spectrum. Recommendations of connecting with other neurodivergent individuals as well as creating spaces for neurodivergent youth to interact with one another, such as in group intervention spaces or autistic school counseling groups, could support the development of neurodivergent community for autistic youth. Clinicians and professionals working with this population should provide psychoeducation to aid families in understanding autism and include adolescents in the feedback sessions. Parent education is an important and well documented therapeutic approach (59), but providers should consider the rights of autistic individuals to know about their own diagnoses and the importance of accurate knowledge about autism for self-understanding. Education about the benefits of parental disclosure, conversations about autism, and accurate knowledge transfer to the autistic individuals could all be included in feedback and parent education materials following a diagnosis.

## 5 Limitations and future directions

We did not explicitly ask about participants’ experiences in relation to their multiple identities (e.g., race, ethnicity, gender), and thus future studies conducted with autistic adolescents should explicitly explore identity across multiple axes. This sample was majority white and included only adolescents from the Washington, D.C. metropolitan area, and thus future studies should seek to recruit a more ethno-racially and geographically diverse sample. Our findings inform how clinicians and parents/families can communicate with autistic adolescents around their autism diagnosis. Given that adolescents spend considerable amounts of time in schools, future research should consider experiences of adolescents in the school environment and generate recommendations for school personnel in supporting positive autistic identity development and autism education.

This study employed a brief semi-structured interview, and responses were similarly brief. Future studies should employ a more comprehensive interview and follow up on brief responses to further explore autistic adolescents’ thoughts and feelings about their diagnoses. This study did not ask about age of diagnosis, time since diagnosis, diagnostic status of family and close friends, integration in neurodivergent community, or whether parents had disclosed the diagnosis to participants, which makes it challenging to draw conclusions about the importance of these factors. Due to the emerging ideas about the impact of these variables, future research should seek to explore these concepts more. While this study included an autistic author and interviewer in the data collection process, autistic individuals were not meaningfully involved in the interview question selection process. Future studies should ensure autistic community partnership throughout the research process.

## 6 Conclusions

Autistic adolescents have diverse, complex, and nuanced thoughts about their autism diagnoses, representing a wide range of autistic identity integration. Adolescents used varied terminology to describe their autism, with some choosing language that deliberately distanced them from other autistic people, and some doubting or denying their autism diagnosis altogether. Others found that their diagnoses helped them understand themselves better or were part of their emerging self-concept. Importantly, several adolescents demonstrated a clear lack of knowledge about autism, sometimes voicing a desire to know more (e.g., asking the interviewer for more information). Most adolescents were engaged in a continual, iterative process of weighing and wrestling with their feelings to make meaning of their autism diagnoses, often comparing the impact of their autism to other co-occurring diagnoses in their day-to-day experiences. Findings illustrate the impact of internalized stigma, importance of neurodivergent community, and knowledge about autism on adolescents’ identification with autism. Our results have implications for families and clinicians to help promote a positive autistic identity and self-acceptance for adolescents. Based on our findings, our research team offers recommendations for ways that providers and families can foster neurodivergent community, neurodiversity-aligned views, and accurate knowledge of autism.

## Data Availability

The raw data supporting the conclusions of this article will be made available by the authors, without undue reservation.
